# The RNAi pathway plays a small part in *Wolbachia*-mediated blocking of dengue virus in mosquito cells

**DOI:** 10.1038/srep43847

**Published:** 2017-03-06

**Authors:** Gerard Terradas, D. Albert Joubert, Elizabeth A. McGraw

**Affiliations:** 1 School of Biological Sciences, Monash University, Clayton VIC 3800, Melbourne, Australia; 2 Institute of Vector-borne Disease, Monash University, Clayton VIC 3800, Melbourne, Australia

## Abstract

*Wolbachia pipientis* is an insect endosymbiont known to limit the replication of viruses including dengue and Zika in their primary mosquito vector, *Aedes aegypti. Wolbachia* is being released into mosquito populations globally in a bid to control the diseases caused by these viruses. It is theorized that *Wolbachia’s* priming of the insect immune system may confer protection against subsequent viral infection. Other hypotheses posit a role for competition between *Wolbachia* and viruses for host cellular resources. Using an *A. aegypti* cell line infected with *Wolbachia*, we tested the effects of targeting siRNAs against the major innate immune pathways on dengue virus loads. We show that while *Wolbachia* infection induces genes in the Toll, JAK/STAT and RNAi pathways, only reduced expression of RNAi leads to a rebound of dengue virus loads in *Wolbachia*-infected cells. The magnitude of the effect explained less than 10% of the total DENV load, demonstrating that blocking must be dependent on other factors in addition to the expression of RNAi. The findings bode well for the long-term stability of blocking given that immunity gene expression would likely be highly plastic and susceptible to rapid evolution.

Arthropod-borne diseases, mainly those transmitted by mosquitoes, are one of the leading causes of mortality in humans, especially in tropical and subtropical areas^1^. Dengue virus (DENV, serotypes 1–4) is a positive single stranded RNA virus of the family *Flaviviridae* and the causative agent of dengue fever, a debilitating illness and the most prevalent of all arthropod-borne diseases worldwide^2^,^3^. Current estimates suggest upwards of 400 million people are at risk of becoming infected annually^4^,^5^. DENVs are transmitted to humans during blood feeding by female *Aedes* mosquitoes: *Aedes aegypti* is the main vector and, to a lesser extent, *Aedes albopictus*^2^,^6^,^7^.

The virus is spreading quickly due to globalization^8^ and climate change^9^, which is allowing *Aedes spp.* to colonise traditionally colder regions. Current vaccines are imperfect^10^,^11^ and there are no effective antivirals. Additionally, the severity of outbreaks appears to be increasing^12^. More recently, Zika virus, also vectored by *A. aegypti*, has re-emerged with devastating health and socioeconomic impact worldwide^13^,^14^. Current strategies for limiting arthropod-borne diseases are heavily dependent on effective vector control^4^. The most novel of these type of approaches relies on the use of an insect bacterial endosymbiont, *Wolbachia*, that has the capacity to limit the replication of arboviruses inside mosquito vectors^15^.

*Wolbachia pipientis* is vertically transmitted by females to their offspring and drives its own spread through insect populations by manipulating host reproductive success to its own advantage^16^. The ability to invade a population and be self-sustaining is hugely appealing with respect to its potential use for biological control. Not native to most of the major insect vectors, *Wolbachia* had to be transinfected from *Drosophila melanogaster* into *A. aegypti*, where it then formed a stably inherited infection^17^. The ability of *Wolbachia* to spread into native *A. aegypti* populations and remain at high frequencies was first demonstrated in Cairns, Australia^18^. *Wolbachia* also has been shown to reduce the replication of a range of pathogens inside insects including viruses, bacteria, nematodes and the malaria parasite^19^,^20^,^21^,^22^. Subsequently, release programs have begun in multiple locations throughout the tropics.

Despite the global scale of *Wolbachia’s* release, the mechanism of *Wolbachia-*based ‘pathogen blocking’ is not well understood. Modulations of essential cellular components such as cholesterol^23^ and host microRNAs^24^, as well as competition between pathogens and *Wolbachia* for limited host resources^21^ have been theorized to underpin blocking. It has also been suggested that the diversity of pathogens blocked by *Wolbachia* could be explained by a *Wolbachia*-mediated host gene modulation leading to an increase in the basal immune activity of the host^21^,^22^. Any subsequent exposure to a pathogen would therefore lead to greater ability to control the assault, in a theory deemed ‘innate immune priming’^25^,^26^.

The primary humoral pathways of the insect innate immune response include Toll, Immune Deficiency (Imd), Janus Kinase-Signal Transducer Activator of Transcription (JAK/STAT) and the exogenous siRNA pathway as part of the RNAi response (Fig. 1)^27^,^28^,^29^,^30^,^31^,^32^,^33^. Additionally, there are cellular responses including the Toll-induced autophagy pathway and melanization cascades^34^,^35^. Each of the pathways has some specificity with respect to type of pathogen targeted, i.e. bacteria, viruses or fungi but several of the pathways are not yet completely defined and there is a growing evidence of overlap between pathways classified as antimicrobial or antiviral^36^,^37^. For example, the Toll and Imd pathways are primarily antibacterial in effect, but Toll is also required for the mosquito’s response to DENV^38^,^39^.

Multiple studies have demonstrated that *Wolbachia* infection increases the basal expression of innate immunity genes^22^,^25^,^26^. In *D. melanogaster, Wolbachia* does not induce the Toll and Imd pathways. Regardless, there is some evidence of *Wolbachia-*mediated blocking when DENV is injected into the fly, suggesting these pathways are not required^40^. Immune activation by *Wolbachia* in the mosquito, however, is stronger and more widespread in terms of genes and pathways affected^26^,^41^,^42^. This difference in the nature of immune activation may also explain the much broader spectrum of pathogen blocking seen in the mosquito including antibacterial and antiviral effects^42^. Few studies have addressed the role of the primary antiviral pathways JAK/STAT and RNAi, or cellular responses like autophagy in regards to *Wolbachia-*mediated blocking in mosquitoes^43^.

The induction of the JAK/STAT pathway has been shown to restrict infection of another flavivirus, West Nile virus, in *Culex* mosquitoes^44^ and the malaria parasite in *Anopheles gambiae*^45^. In *A. aegypti*, the antiviral function of the pathway is conserved with high activation levels of JAK/STAT limiting DENV replication. The effectors of this pathway, however, are poorly characterized^32^. RNAi is considered one of the major antiviral pathways, shown to limit DENV, chikungunya and Sindbis viruses in *A. aegypti*^29^,^46^,^47^ but seems less important for pathogen blocking in insects with native *Wolbachia* infections^48^,^49^. The RNAi pathway is initiated with the recognition and cleavage of viral double stranded RNA (dsRNA) into siRNAs that then operate through cellular machinery to degrade viral ssRNA. Lastly, the cellular responses of autophagy^33^,^50^ and apoptosis also have some antiviral relevance^51^,^52^.

In this study we investigated which components of the mosquito innate immune response are both primed by *Wolbachia* and are essential to induce DENV blocking. We focused on each of the above mentioned major pathways, selecting genes to represent pathways that were either starting points in a signaling cascade, like *MyD88* (Toll) and *FADD* (Imd), or because they serve as effectors, like *vir-1* (JAK/STAT), argonaute-*2 (AGO2*, RNAi) and *APG5* (autophagy). We then examined the functional role of each associated pathway in DENV control in *A. aegypti* cells by manipulating gene expression via targeted RNAi techniques in both *Wolbachia*-infected and *Wolbachia*-free cells. We predicted that if genes were uninvolved in *Wolbachia*-mediated effects, reducing their expression via siRNA treatment should have little impact on DENV loads in *Wolbachia-*infected cells. In contrast, if the *Wolbachia*-mediated blocking was reliant on the activity of particular genes, DENV loads should rebound after siRNA treatment. We were particularly interested in genes exhibiting this pattern, whose basal expression was also enhanced by *Wolbachia*.

## Results

To gauge the relative contribution of each of the immune pathways to *Wolbachia*-mediated DENV blocking, candidate genes for each pathway were knocked down in an *A. aegypti* embryonic cell line infected with *Wolbachia* and a tetracycline treated version of the same line (Aag2 ± *w*Mel) that served as a *Wolbachia*-free control. Reductions in gene expression were confirmed 18 h post-transfection. Cell lines were then challenged with a DENV-2 strain to assess whether reduced activity of each immune pathway lead to a corresponding increase in DENV load at 5 days post-infection. In two cases (*MyD88* and *FADD*) where selected genes were early in the pathway, we also assessed whether expression changes were carried through to effectors. To assess whether the different pathways have an additive effect on inhibiting DENV replication, we performed consecutive treatments with siRNA for pairs of genes prior to the challenge with DENV-2. We first showed that transfection with a non-*Aedes* targeted siRNA did not alter gene transcription levels so it could be used as a transfection control across samples (Fig. S1). We also demonstrated that single and successive paired siRNA treatment had no effect on *Wolbachia* densities (Fig. S2).

### Toll and Imd

**Figure 2:**
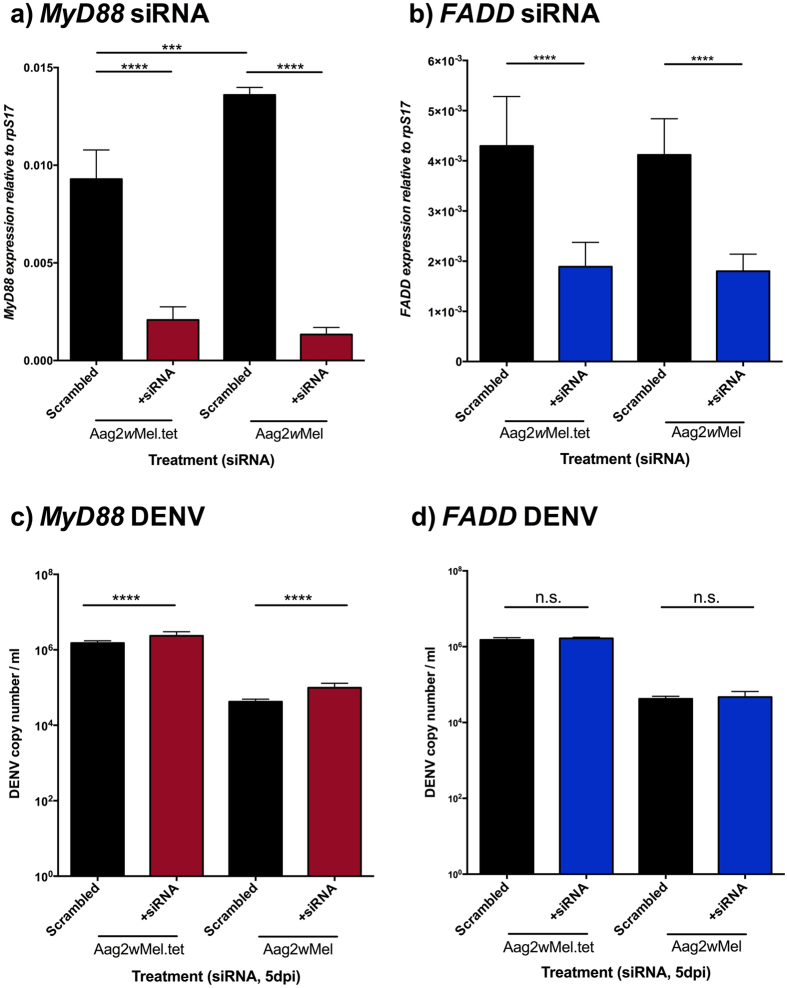
Knockdown of antibacterial pathways. Cell knockdown in *Wolbachia*-infected or tetracycline-treated Aag2 for (**a**) *MyD88* (Toll) and (**b**) *FADD* (Imd). Gene expression was normalized to *A. aegypti* housekeeping gene rpS17. Graphs (**c,d**) correspond to DENV loads after cells were challenged with DENV-2 and collected at 5dpi. All graphs show medians with interquartile ranges (n = 12 per treatment). Black columns depict scrambled controls. Significance is based on post-hoc comparisons following ANOVAs on logarithmic transformed data. ***p < 0.001; ****p < 0.0001.

Both *Wolbachia* infection (F = 10.23, df = 1, p = 0.003) and siRNA treatment (F = 611.94, df = 1, p < 0.0001) had significant effects on *MyD88* expression. Posthoc comparisons demonstrated reductions in *MyD88* expression (Fig. 2a) between the treatment and scrambled control for both Aag2*w*Mel.tet (t = 20.03, df = 20, p < 0.0001) and Aag2*w*Mel (t = 14.52, df = 20, p < 0.0001). Concurrent reductions in expression of genes downstream in the Toll pathway demonstrate the generality of the effect (Fig. S3). A direct comparison of the expression levels in the scrambled treatment across lines revealed differences (t = 4.65, df = 20, p = 0.0002) that may be explained by *Wolbachia* infection as well as effects of the antibiotic treatment or drift in the post antibiotic passaging period. This reinforces the need to examine fold changes in expression after siRNA treatment relative to the scrambled control to correct for line effects. This difference may also highlight up-regulation by *Wolbachia* of immunity genes in keeping with the immune priming hypothesis^25^. In comparison, relative to scrambled controls, the magnitude of the reduction in *MyD88* expression was roughly 4.5-fold in the Aag2*w*Mel.tet and 9-fold in Aag2*w*Mel. In the case of FADD expression, siRNA treatment (F = 86.73, df = 1, p < 0.0001) had a significant effect on expression but not *Wolbachia* infection (F = 1.07, df = 1, p = 0.308). *FADD* expression levels were similarly reduced in Aag2*w*Mel.tet (t = 6.46, df = 21, p < 0.0001) and Aag2*w*Mel (t = 6.32, df = 19, p < 0.0001) after siRNA treatment (Fig. 2b). The knock down was also conferred to other genes downstream in the Imd pathway (Fig. S3). The achieved reduction of *FADD* expression in both cell lines relative to scrambled controls was ~2.3-fold.

After 18 h of targeted gene knockdown, infection with a DENV-2 strain was performed and cells were then collected at 5 days post infection (dpi). After Toll modulation, there was a significant effect of both the siRNA treatment (F = 75.66, df = 1, p < 0.0001) and *Wolbachia* infection (F = 1606.62, df = 1, p < 0.0001) on DENV load (Fig. 2c). The results show that the siRNA treatment leads to increases in DENV loads and *Wolbachia* infection leads to reductions in DENV load. There was also a slight significant interaction between *Wolbachia* status and siRNA treatment on DENV load (F = 5.1, df = 1, p = 0.03) as the magnitude of impact on DENV load was greater in Aag2*w*Mel than in Aag2*w*Mel.tet. These represent only 3.0 and 7.4% increase in the DENV load relative to scrambled controls for *Wolbachia*-free and *Wolbachia*-infected, respectively. It is unclear whether these differences are large enough to be biologically meaningful. When challenging *FADD*-knocked down cells (Fig. 2d), DENV loads were affected by *Wolbachia* infection status (F = 360.25, df = 1, p < 0.0001) as expected. There was however no effect of siRNA treatment (F = 1.69, df = 1, p = 0.873).

Our results point to Toll having an important role in *A. aegypti’s* immunity against DENV, but the pathway is not essential to explain the protective phenotype conferred by *Wolbachia,* as reported previously^40^ in *Drosophila*. We also do not see evidence of Imd pathway involvement in DENV protection, since inactivation of the pathway does not lead to an increase in DENV loads and *Wolbachia* infection does not affect its expression.

### JAK/STAT

**Figure 3:**
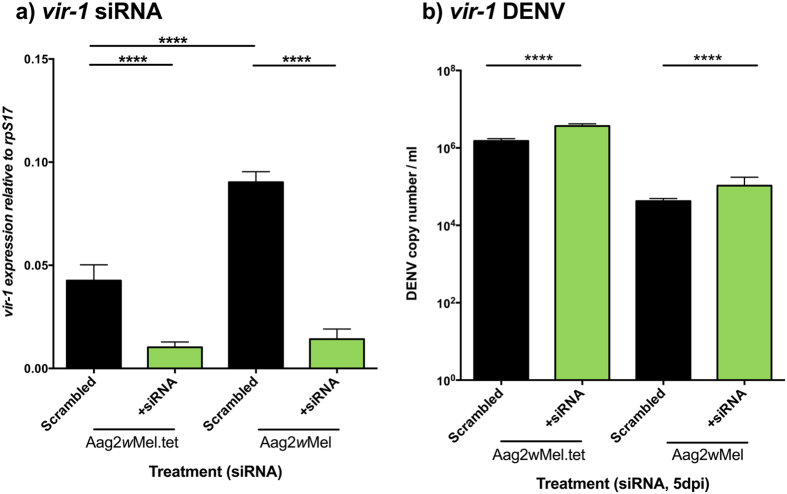
Knockdown of the JAK/STAT pathway. (**a**) *vir-1* gene (JAK/STAT) knockdown in *Wolbachia*-infected or tetracycline treated Aag2 cells. Gene expression was normalized to *A. aegypti* housekeeping gene rpS17. (**b**) DENV loads after knock down and challenge with DENV-2. Graphs show medians with interquartile ranges (n = 12 per treatment). Black columns depict scrambled controls. Significance is based on post-hoc comparisons following ANOVAs on logarithmic transformed data. ****p < 0.0001.

Both *Wolbachia* infection (F = 80.31, df = 1, p < 0.0001) and siRNA treatment (F = 355.13, df = 1, p < 0.0001) had significant effects on *vir-1* expression (Fig 3a). A direct comparison of the expression levels in the scrambled treatment across lines revealed *Wolbachia*-associated increases in expression of *vir-1* (t = 8.63, df = 19, p < 0.0001). The siRNA treatment produced a significant decrease in expression of *vir-1* for both Aag2*w*Mel.tet (t = 15.26, df = 20, p < 0.0001) and Aag2*w*Mel (t = 12.15, df = 20, p < 0.0001). In comparison, relative to scrambled controls, the magnitude of the reduction in *vir-1* expression was roughly 4.2-fold in the Aag2*w*Mel.tet and 6-fold in Aag2*w*Mel. We then tested if DENV levels were affected by the siRNA treatment and the *Wolbachia* infection status (Fig. 3b). There was a significant effect of the siRNA treatment (F = 18.88, df = 1, p < 0.0001) and of the *Wolbachia* infection (F = 337.75, df = 1, p < 0.0001). According to the results, the siRNA treatment increases DENV loads, whereas the presence of *Wolbachia* leads to a reduction in DENV, as expected. These represent only 5.8 and 7.9% increases in the DENV load relative to scrambled controls for *Wolbachia*-free and *Wolbachia*-infected, respectively. Importantly, the reduction of *vir-1* expression in Aag2*w*Mel does not lead to a recovery of DENV toward loads seen in *Wolbachia* uninfected untreated cells.

### RNAi (Exogenous siRNA pathway)

**Figure 4:**
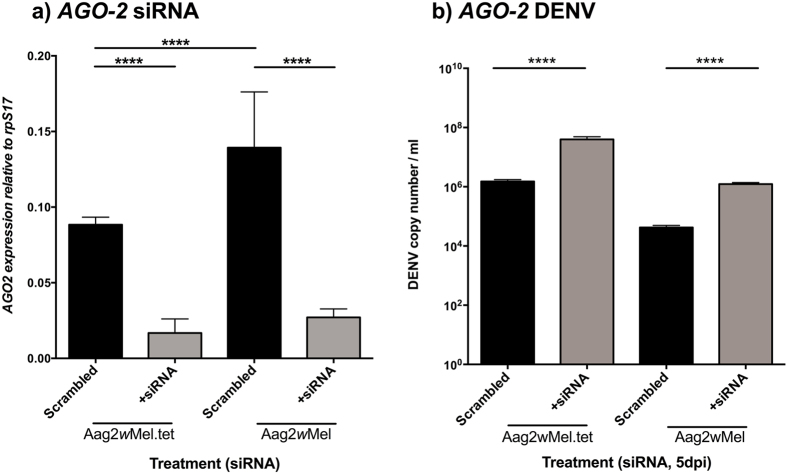
Knockdown of RNAi. (**a**) *AGO2* gene (RNAi) knockdown in *Wolbachia*-infected or tetracycline treated Aag2 cells. Gene expression was normalized to *A. aegypti* housekeeping gene rpS17. (**b**) DENV loads after knock down and challenge with DENV-2. Graphs show medians with interquartile ranges (n = 12 per treatment). Black columns depict scrambled controls. Significance is based on post-hoc comparisons following ANOVAs on logarithmic transformed data. ****p < 0.0001.

Both *Wolbachia* infection (F = 33.88, df = 1, p < 0.0001) and siRNA treatment (F = 264.75, df = 1, p < 0.0001) had significant effects on *AGO2* expression (Fig. 4a). A direct comparison of the expression levels in the scrambled treatment across lines revealed *Wolbachia*-associated increases in expression of *AGO2* (t = 6.29, df = 19, p < 0.0001). Targeted siRNA caused a decrease in *AGO2* expression levels on both Aag2*w*Mel.tet (t = 11.68, df = 19, p = 0.0033) and *w*Mel-infected (t = 14.62, df = 19, p < 0.0001) lines. In comparison, relative to scrambled controls, the magnitude of the reduction in *AGO2* expression was roughly 4.2-fold in the Aag2*w*Mel.tet and 5.3-fold in Aag2*w*Mel. When testing for effects on DENV loads after gene knockdown (Fig. 4b), both the effects of siRNA treatment (F = 1506, df = 1, p < 0.0001) and *Wolbachia* infection (F = 1667, df = 1, p < 0.0001) were significant. Similar to other genes tested, siRNA treatment caused DENV loads to increase whereas *Wolbachia* infection limited DENV replication. These increases in DENV load are on the order of 18.0 and 24.0% relative to scrambled controls for *Wolbachia*-free and *Wolbachia*-infected, respectively. These higher fold changes than previous genes demonstrate the importance in general of AGO2 for DENV control. The differential between the two lines (6%) represents the extra increase in DENV load that is due to *Wolbachia* via RNAi interactions.

Our results suggest that the exogenous siRNA pathway of those examined in the humoral response is the main controller of DENV replication in *Aedes aegypti*. When comparing specific antiviral pathways, the suppression of JAK/STAT through knockdown of *vir-1* doesn’t affect DENV loads as much as inactivation of the exogenous siRNA pathway through knockdown of *AGO2*. The presence of *Wolbachia* increases basal *AGO2* gene levels, which have been proven crucial for DENV control. We hypothesize that *Wolbachia*-mediated blocking of DENV is in part utilizing the exogenous siRNA pathway through up-regulation of its components to control DENV replication. This effect however explains less than 10% of *Wolbachia’s* ability to limit DENV.

### Autophagy

**Figure 5:**
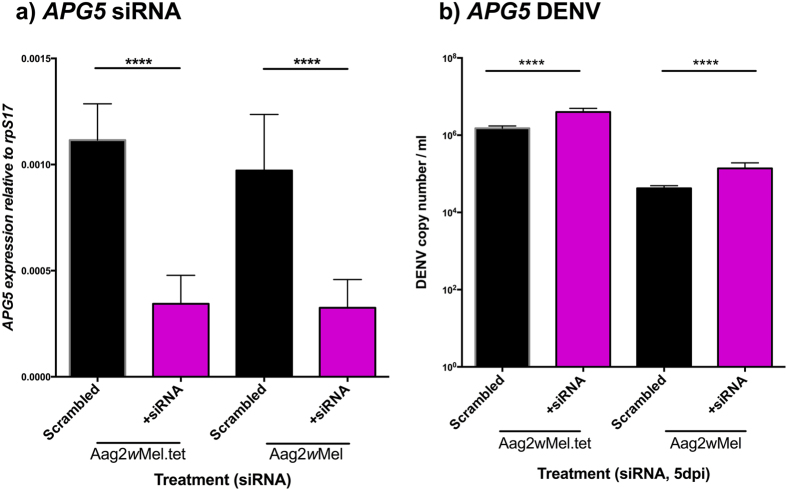
Knockdown of autophagy. (**a**) *APG5* gene (autophagy) knockdown in *Wolbachia*-infected or tetracycline treated Aag2 cells. Gene expression was normalized to *A. aegypti* housekeeping gene rpS17. (**b**) DENV loads after knock down and challenge with DENV-2. Graphs show medians with interquartile ranges (n = 12 per treatment). Black columns depict scrambled controls. Significance is based on post-hoc comparisons following ANOVAs on logarithmic transformed data. ****p < 0.0001.

There was a significant effect of siRNA treatment (F = 132.18, df = 1, p < 0.0001) but not *Wolbachia* infection (F = 0.60, df = 1, p = 0.44) on APG5 expression (Fig. 5a). Successful knockdown of *APG5* was achieved in Aag2*w*Mel.tet (t = 8.14, df = 21, p < 0.0001) and Aag2*w*Mel (t = 8.61, df = 18, p < 0.0001), reducing *APG5* levels ~2.8-fold in both cell lines compared to scrambled control. A direct comparison of the expression levels in the scrambled treatment across lines also revealed no effect of *Wolbachia* infection on expression of *APG5* (t = 0.96, df = 19, p < 0.34). There was a significant effect of siRNA treatment (F = 142.55, df = 1, p < 0.0001) and *Wolbachia* status (F = 1365, df = 1, p = 2.75e^−7^) on DENV load (Fig. 5b). Our results show that siRNA treatment leads to an increase in DENV load and *Wolbachia* presence reduces DENV. In comparison, relative to scrambled controls, the magnitude of the reduction in *APG5* expression was roughly 6.3-fold in the Aag2*w*Mel.tet and 10.0-fold in Aag2*w*Mel.

Even though DENV loads are increased with the suppression of *APG5*, the presence of *Wolbachia* does not modulate *APG5* levels and the lines respond similarly to siRNA treatment. Altogether, it allows us to conclude that *Wolbachia*-based protection is not reliant on autophagy. This non-modulation of its expression levels indicates that autophagy is acting independently of the presence of a *Wolbachia* infection, but is probably an important factor in the *A. aegypti* general immune response against dengue virus.

### Stacking of Toll, JAK/STAT and RNAi

Sequential delivery of siRNAs for individual genes followed by *AGO2* was necessary to test combined effects of pathways, as the efficacy of the siRNA treatment itself relies on a functional RNAi response. We first determined whether the knockdown of each individual gene was affected by the presence of the second siRNA. In all cases, save *vir-1* in Aag2*w*Mel only, the knockdown for each gene was the same between the single gene and stacked approach (Fig. S4), indicating a lack of interference or overlap between siRNAs. As previously reported, we found a *Wolbachia*-mediated up-regulation for three genes belonging to three different innate immune pathways. The genes were *MyD88* (Toll; t = 3.461, df = 22, p = 0.0022), *vir-1* (JAK/STAT; t = 4.257, df = 22, p = 0.0003) and *AGO2* (exogenous siRNA; t = 4.495, df = 22, p = 0.0002).

**Figure 6:**
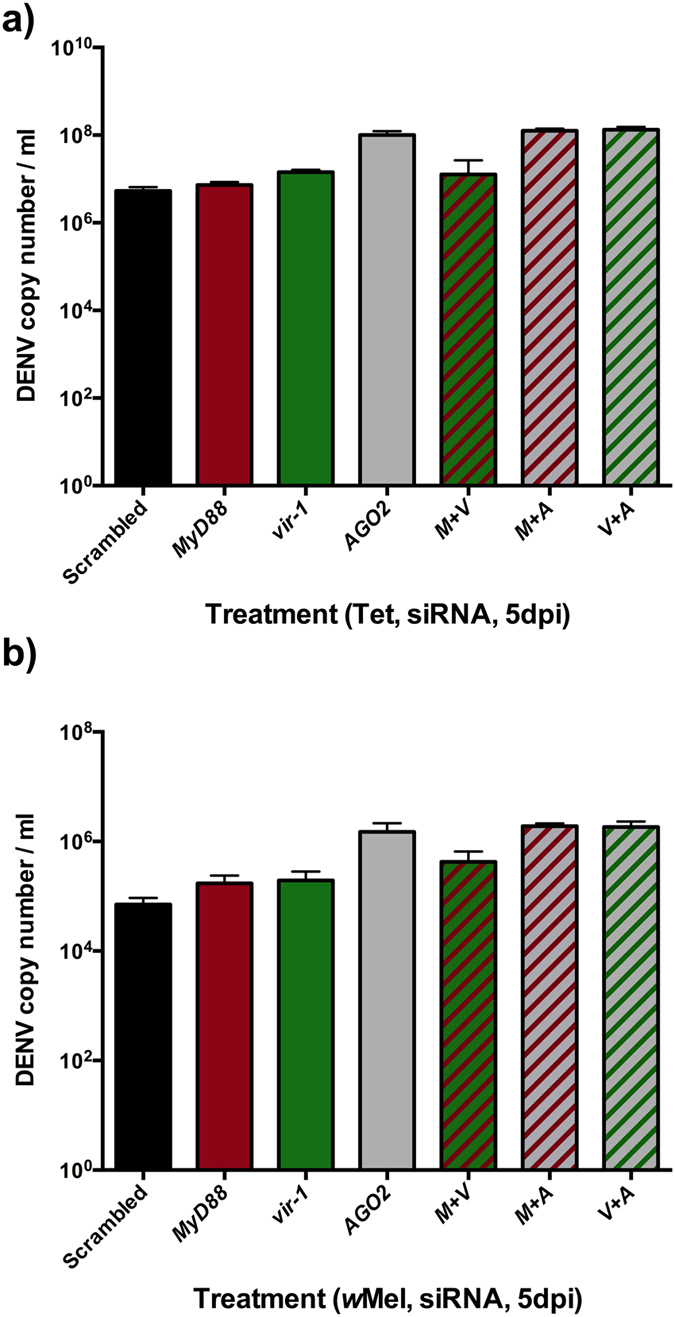
Knockdown of stacked *MyD88, vir-1* and *AGO-2*. DENV load in response to single gene (solid bars) and stacked gene knockdowns (hatched bars) for (**a**) Aag2*w*Mel.tet and (**b**) Aag2*w*Mel. Black columns depict scrambled controls. Graphs show medians with interquartile ranges (n = 12 per treatment). Statistical significance reported in Table 1.

Also shown previously in the single gene siRNA assays, the greatest increase in DENV load was for *AGO2*-treated samples (Fig. 6) for both *Wolbachia*-free (t = 28.04, df = 22, p < 0.0001) and infected line (t = 13.98, df = 21, p < 0.0001). In each case the control comparisons of single gene siRNA treatments versus the scrambled controls recapitulated the findings in the single gene assays. There was no effect of sequential treatment of siRNAs for *vir-1* and *MyD88* together compared to *vir-1* alone on DENV load (Fig. 6, Table 1). In contrast, the stacking of *MyD88* and *vir-1* siRNAs each with *AGO2* produced greater DENV loads than for *AGO2* alone, demonstrating the contributory role of these pathways in DENV control. A marginally significant interaction for both of these comparisons *AGO2* resulted from the slightly larger effect of siRNA treatment in *Wolbachia* infected cells compared to *Wolbachia*-free. The results of the stacking assays demonstrate the greater importance of *AGO2* than *MyD88* and *vir-1* in DENV control but also in *Wolbachia*-mediated blocking. The contributions from the latter two pathways, while small, are additive.

## Discussion

In this study we aimed to determine if any of the major innate immunity pathways in mosquitoes plays a role in *Wolbachia-*mediated DENV blocking. Using siRNA against single and pairs of genes, we reduced the transcription of key genes representing each of 5 immunity pathways in mosquito cells and assessed the impact of gene knockdown on DENV load. By comparing the responses of *Wolbachia*-infected to *Wolbachia*-free cells, we were able to specifically determine which genes were involved not only with anti-DENV responses, but contributing specifically to *Wolbachia*-mediated effects. The Toll, JAK/STAT and RNAi pathways all demonstrated increased basal expression in response to *Wolbachia* infection. When expression of the RNAi pathway was reduced, however, DENV loads in *Wolbachia*-infected cells rebounded, suggesting that *Wolbachia*-mediated blocking was in part reliant on the action of RNAi. The magnitude of this effect was small however, explaining less than 10% of the total DENV load. The differential involvement of genes in DENV control across the two lines was also seen for Toll and JAK/STAT, with greater DENV load increases seen in the Aag2*w*Mel cell line compared to the tetracycline-treated line. These additional pathways therefore provide a small but significant contribution to the blocking conferred by RNAi. The additive nature of these gene contributions was further confirmed with the stacking assays.

In insects, RNAi plays a key role in antiviral defense. We focused on components of the exogenous siRNA pathway, whose primary function is the cleavage of dsRNA^53^. Briefly, exogenous dsRNA molecules are recognized and cleaved into smaller fragments (siRNAs) by a dsRNA-specific RNAse (Dicer). Then, siRNAs destroy their complementary mRNA targets by binding and guiding the complex to the argonaute protein that carries out endoribonucleic cleavage^54^. Because the silencing activity of siRNAs is responsive to the particular agent infecting the cell at any one time, it has broad efficacy against diverse viruses. Even though DENV is a (+) ssRNA virus, detectable amounts of dsRNA are created as replicative intermediates, similar to other flaviviruses^55^. Several studies have demonstrated the involvement of RNAi responses in regulating arboviruses including chikungunya, DENV, yellow fever or o’nyong-nyong inside the vector^46^,^47^,^56^,^57^.

Interestingly, in *Drosophila*, RNAi is not essential to achieve *Wolbachia*-mediated blocking from Drosophila C Virus^48^ and nor is it needed for control of Semliki Forest Virus in *Drosophila* cells infected with *Wolbachia*^43^. The fly and the mosquito may differ, however, with clear evidence of a much stronger and more far reaching immune response to *Wolbachia* in that latter^42^,^58^. Studies across a range of insect species suggest that older and more established *Wolbachia*:host associations may have lower *Wolbachia* densities, contracted tissue distributions and fewer effects on the immune response^59^,^60^,^61^,^62^. That *Wolbachia* may be causing a greater reaction in the recently infected *A. aegypti* is in keeping with co-evolutionary theories for novel host:pathogen pairings^63^. Additionally, mosquitoes/mosquito cells themselves may have histories of adaptation to native viruses that are not seen when non-native hosts like *Drosophila* are infected.

JAK/STAT has also been proposed as a major pathway involved in antiviral protection^28^,^32^. Our data supports previous studies suggesting that RNAi is more important for DENV control than JAK/STAT^46^. This difference between the two main antiviral pathways may stem from their mode-of-action. RNAi pathways are triggered by intracellular exogenous dsRNA^64^ whereas the activation of JAK/STAT is dependent on the binding of secreted ligands following pathogen recognition^36^. JAK/STAT’s antiviral mode of action remains unknown, though it is thought to be complex and versatile^65^. Moreover, DENV also suppress many signaling pathways. For example, the viral protein NS4B causes inhibition of the IFN pathway (JAK/STAT is involved in mammal type I interferon signaling^66^) by preventing STAT1 phosphorylation and activation, as well as its transport to the nucleus^67^. Similarly, NS5 has been shown to inhibit human STAT2 phosphorylation^68^. In mosquitoes, inhibition of immune signaling pathways Toll, Imd and JAK/STAT has been shown in the DENV-related Semliki Forest Virus^69^.

It is unclear how *Wolbachia* may be modulating the expression of either the RNAi or JAK/STAT pathways. Because RNAi acts intracellularly, there may be greater opportunities for the symbiont to manipulate its expression than for pathways like JAK/STAT. However, while cross talk has been demonstrated between JAK/STAT and Toll^70^, such interactions have not yet been shown between RNAi and other pathways that may respond directly to bacterial effectors. A previous study has shown that *Wolbachia* has the capacity to affect intracellular localization of AGO-1, a member of the miRNA pathway within RNAi^71^. It is not clear how this change is mediated, but it has capacity to affect the host’s immune response to pathogens.

Several aspects of the study’s design may limit the scope of its interpretation. First, as with any siRNA approaches, the ability to detect phenotypic effects is dependent on the strength of silencing. In almost all cases transcription was reduced by ~75% but we cannot rule out that additional effects might have emerged if we had reduced the gene expression further. Next, we have utilized the standard approach of creating a *Wolbachia*-free line by tetracycline treatment. Although few passages were allowed for treatment with antibiotics and for recovery, it is possible that some genetic drift occurred between the treatment and control lines. Regardless, the comparisons via a scrambled control intermediate should mitigate such issues. Creation of a newly *Wolbachia*-infected line where the original recipient line serves as the wildtype is also likely to lead to drift given the number of reinfection events and also selection that must commonly be employed to get a highly infected cell line^72^. Last, the approach taken here is highly reductionist. Blocking in adult mosquitoes is likely to be more complicated given the diversity of cell and tissue types and their potential to vary with respect to immune activity^73^. There is also likely, yet to be discovered, avenues of cross talk between immune pathways. More generally, these are complex interactions involving three organisms and their genomes. While the results can speak directly to the involvement of immune priming, our ability to estimate its proportional involvement relative to other mechanisms is limited.

*Wolbachia* is being released in a number of sites throughout the tropics as a possible biocontrol agent against viruses vectored by *A. aegypti*, including DENV and Zika^15^,^74^. Understanding mechanistically how *Wolbachia* restricts pathogen replication is key for assessing the long-term evolutionary stability of pathogen blocking in vector populations. Our findings show that increased expression of a gene in the mosquito’s antiviral response confers a small amount of DENV blocking in cells. Given the breadth of involvement of RNAi in protection against a range of arboviruses, this is likely to be true for Zika and other viruses vectored by *A. aegypti*. The concern, if blocking were heavily reliant on this immune reaction and not other factors, is the plasticity of gene expression as well as its capacity to evolve in response to pathogens^75^. In the field, mosquitoes may evolve tolerance to *Wolbachia* and limit the need to mount a costly immune response to the symbiont^76^. The vector may also evolve resistance, limiting *Wolbachia* densities or tissue distributions, as tends to be seen in native hosts such as *Drosophila*^42^,^61^,^62^,^73^,^77^. Field release populations of *A. aegypti* are being monitored to assess the stability of blocking and strategies are being developed for dealing with emerging resistance^78^. This study suggests at least that the risk reduced blocking efficacy due to a rapidly evolving immune response is low.

## Materials and Methods

### Cell line maintenance

The *w*Mel strain was transinfected from *D. melanogaster* into the immune-competent *A. aegypti* cell line Aag2^79^,^80^ using the shell vial technique, as previously performed for other mosquito cell lines^72^,^81^. The Aag2*w*Mel cell line was serially passaged and checked for *Wolbachia* infection using quantitative PCR (qPCR)^18^ and fluorescent *in situ* hybridization (FISH) against *Wolbachia-*specific 16S rRNA probe^82^. The control *w*Mel uninfected line (Aag2*w*Mel.tet) was obtained after three successive passages in the presence of tetracycline treatment at 10 mg/ml. The complete absence of *Wolbachia* was also confirmed using FISH and qPCR. Both cell types were routinely passaged in filtered complete media: a 1:1 mixture of Schneider’s media (Life Technologies) and Mitsuhashi-Maramorosch (MM), supplemented with 10% heat-inactivated FBS (Life Technologies) and 1% Penicillin/Streptomycin (Life Technologies). Cells were reared in an incubator at 25 °C.

### Virus

Dengue virus serotype-2 strain (ET-300; GenBank: EF440433.1) was isolated from a patient in East Timor-Leste in 2000 and passaged in C6/36 cells prior to experimental use. C6/36 cells were continuously kept in RPMI 1640 media (Life Technologies, Carlsbad, CA) supplemented with 10% heat-inactivated fetal bovine serum (FBS, Life Technologies), 1% Glutamax (Life Technologies), and 25 mM HEPES buffer (Sigma-Aldrich, St. Louis, MO). Cells were kept in a non-humidified incubator at 25 °C for optimal growth. Cells were allowed to grow to an 80% confluent monolayer prior to virus inoculation for 2 h and then maintained in 2% FBS media. Virus was collected at 7 days post-infection by harvesting the cell culture supernatant and centrifuged at max speed for 15′ at 4 °C. Virus was aliquoted and stored at −80 °C until use. Viral stocks were titrated using plaque assays and dengue copies quantified via qPCR. ET300 viral stocks were diluted in serum-free RPMI media to a concentration of 4 × 10^5^ plaque forming units per milliliter before experimental use.

### ***Wolbachia*** density

*Wolbachia* densities were measured in Aag2*w*Mel after every passage and Aag2*w*Mel.tet lines were assessed in parallel to confirm their uninfected status. We also assessed the effect of siRNA treatment on *Wolbachia* densities. Taqman^®^ multiplex qPCR was performed to detect the *w*Mel strain levels using primers for the *Wolbachia WD0513* gene^83^ relative to the mosquito housekeeping mosquito gene *rpS17* 84 in a LightCycler480 instrument (Roche Applied Science, Switzerland). The primers used are listed in Table 2. Each multiplexed qPCR was run in triplicate and consisted of a 2x LightCycler480 Probes Master reaction mix, 10 μM *rpS17* and *w*Mel-IS5 primers, probes and 1.5 μl of DNA template in a total volume of 10 μl, as stated in the manufacturer’s protocol. Ratios of *w*Mel-IS5 to *rpS17* were obtained following the ∆∆Ct method^85^.

### siRNA transfection

Aag2*w*Mel and Aag2*w*Mel.tet cells were seeded the day before transfection in a flat bottom Greiner 96-well plate (Sigma-Aldrich) at a 70–80% confluence. The following day, three different treatments were applied to the cells in a serum-free environment: Mock transfected, Scrambled siRNA at 10 μM and *gene of interest* (GOI) siRNA at 10 μM. Custom siRNAs targeting *A. aegypti* immune genes were manufactured by Sigma-Aldrich. All siRNA treatments were performed with the addition of Lipofectamine RNAiMAX (Sigma-Aldrich) reagent, and transfected altogether according to manufacturer’s protocol. The single gene assays (n = 6 replicate wells per treatment) were replicated a second time and pooled (n = 12). For stacking experiments, (n = 12 replicate wells per treatment) the second GOI siRNA was applied to the sample 18 h after the first siRNA treatment, performed as stated above.

### RNA extraction and cDNA synthesis

Cell RNA was extracted from cells 18 to 36 h hours post transfection using the Nucleospin 96 RNA kit (Machery-Nagel, Germany), following modified manufacturer’s protocol. cDNA synthesis reactions were performed using SuperScript^®^ III Reverse Transcriptase (Invitrogen) and contained 12.5 μl of RNA template, 1 μl of random primers (RP, 125 ng/μl), 1 μl of deoxynucleotides (dNTPs, 2.5 mM), dithiothreitol (DTT), 5X buffer and enzyme as per kit instructions, in a total volume of 20 μl. Reactions were carried out in a C1000™Thermal Cycler (Bio-Rad) with the cycling regime: 65 °C for 5 min followed by 10 min at 25 °C, 50 min at 50 °C, 10 min at 75 °C.

### Selection of candidate immunity genes

Candidate genes were selected for each pathway of interest that met the following criteria: they had to be required for the function of the pathway, be sufficiently transcribed and the ability to be knocked down efficiently in our cell model. Refer to Table 2 for chosen candidates and associated primer sequences with gene IDs and function. All primers were designed using the open-source Primer3 software^86^. For candidates involved upstream we confirmed that expression of downstream genes (Toll and Imd) was reduced after targeted knockdown to assure complete inactivation of the pathway.

### Immunity gene quantitative PCR analysis

Gene expression was measured using SYBR^®^ Green I Master (Roche) according to manufacturer’s protocol. All runs were performed in duplicate using a 10 times dilution of the cDNA. The temperature profile used is as follows: one cycle at 95 °C for 5 min followed by 45 amplification cycles of 95 °C for 10 sec, 60 °C for 10 sec and 72 °C for 10 sec, followed by a melting curve analysis after the last cycle. In all qPCR analyses, GOI were normalized to the housekeeping gene *rpS17,* run in parallel. GOI to housekeeping gene ratios were obtained for each biological sample using the aforementioned ∆∆Ct method^85^.

### DENV infection and quantification

DENV infections were performed 18 h post-transfection with siRNA. Cells were washed with PBS before and after the virus inoculation at a DENV-2 multiplicity of infection (MOI) of 0.5. At this MOI DENV*-*blocking in *w*Mel is most clearly seen as per pilot studies (data not shown). The viral inoculum was removed 2 h post-infection and cells were grown in complete media containing 2% FBS. DENV was quantified 5 days post-infection by collection of 20 μl supernatant and mixed 1:1 with 20 μl squash buffer (10 mM Tris base, 1 mM EDTA, 50 mM NaCl and 0.25 μl proteinase K). They were then incubated in a C1000™Thermal Cycler (Bio-Rad, California USA) at 56 °C for 5 min, then 98 °C for 5 min for the simultaneous isolation of RNA and DNA. The RNA/DNA was subsequently used for the absolute quantification of DENV-2 via qPCR using a standard curve, as described previously^87^.

One-step quantitative PCR was performed using TaqMan^®^ Fast Virus 1-step Master Mix (Roche) in a total 10 μl, following manufacturer’s instructions.

The primer sequences used for the detection of DENV were as described previously^87^. The thermal profile was as stated previously for qPCR analysis, with the addition of 10 min incubation retrotranscription step at 50 °C followed by 20 sec at 95 °C for RT inactivation at the start of the run.

### Data analysis

qPCR reactions were run in duplicate and samples that failed to amplify for at least one replicate were removed. Statistics were carried out using IBM SPSS Statistics (v23) and R software (R Development Core Team (2008), Vienna, Austria). Gene expression ratios and dengue loads were both log transformed prior to analysis. Two-way ANOVAs were performed testing for the effects of *Wolbachia* infection (presence/absence), siRNA treatment (+/−) and replicate (1, 2) as factors on gene expression or DENV load. At no point was ‘replicate’ significant and so reported statistics focus only on main effects. Interactions are reported only when significant. Post hoc comparisons were employed multiple tests accounted for using a Bonferroni correction, leading to adjusted p-values of 0.025 and 0.017, when 2 or 3 comparisons were made, respectively. All DENV loads were reported on a log scale given the spread of values.

## Additional Information

**How to cite this article**: Terradas, G. *et al*. The RNAi pathway plays a small part in *Wolbachia*-mediated blocking of dengue virus in mosquito cells. *Sci. Rep.* **7**, 43847; doi: 10.1038/srep43847 (2017).

**Publisher's note:** Springer Nature remains neutral with regard to jurisdictional claims in published maps and institutional affiliations.

## Supplementary Material

Supplementary Figures

## Figures and Tables

**Figure 1:**
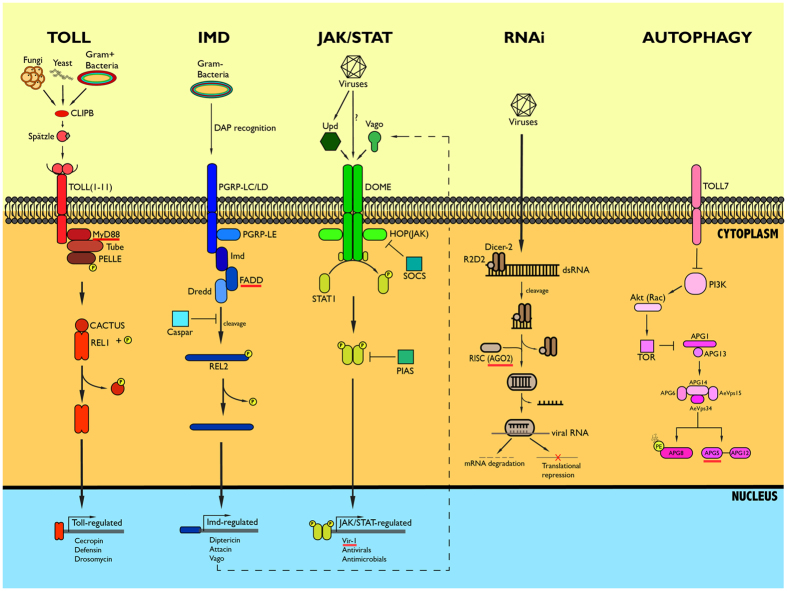
The main *Aedes aegypti* innate immune pathways, The Toll, Imd, JAK/STAT, RNAi and Autophagy. All genes shown correspond to the *A. aegypti* relationships and nomenclature. Underlined genes are targeted with siRNA in this study.

**Table 1:** Analysis of Variance table investigating single gene siRNA vs scrambled control and sequential treatment siRNA versus single gene treatments on DENV for *Wolbachia*-infected and *Wolbachia*-free lines.

Comparison	Factors	F value	df	p
Scrambled vs *MyD88*	siRNA treatment	12.31	1	0.001
	*Wolbachia* (+/−)	526.62	1	<0.001
	siRNA*Wolbachia	9.46	1	0.004
Scrambled vs *vir-1*	siRNA treatment	97.82	1	<0.001
	*Wolbachia* (+/−)	467.72	1	<0.001
	siRNA*Wolbachia	91.26	1	<0.001
Scrambled vs *AGO2*	siRNA treatment	154.73	1	<0.001
	*Wolbachia* (+/−)	180.91	1	<0.001
	siRNA*Wolbachia	145.59	1	<0.001
*vir-1* vs *MyD88* + *vir-1*	siRNA treatment	0.898	1	0.35
	*Wolbachia* (+/−)	113.28	1	<0.001
	siRNA*Wolbachia	0.527	1	0.47
*AGO2* vs *AGO2* + *MyD88*	siRNA treatment	4.84	1	0.033
	*Wolbachia* (+/−)	478.75	1	<0.001
	siRNA*Wolbachia	4.28	1	0.045
*AGO2* vs *AGO2* + *vir1*	siRNA treatment	7.12	1	0.011
	*Wolbachia* (+/−)	353.1	1	<0.001
	siRNA*Wolbachia	6.72	1	0.013

**Table 2:** Candidate genes and associated primers for each pathway.

Gene ID	Pathway	*Aedes* gene name	Direction	Sequence (5′-3′)	Tm	Gene function
AAEL017251	RNAi	*argonaute-2*	Fw	ACAACAGCAACAATCCCAGA	60	Catalytic compound of RISC, mRNA cleavage
			Rv	GTGGACGTTGATCTTGTTGG	60	
AAEL002286	Autophagy	*APG5*	Fw	CCAGGACTTGTTGGAGGACT	56	Autophagosome elongation
			Rv	GTCCGGATAGCTGAGGTGTT	56	
AAEL000627	Toll	*cecropin-A*	Fw	CCATGGCTGTTCTTCTCCTGA	60	Antimicrobial peptide
			Rv	GGCGGCATTGAAAACTCGTT	60	
AAEL004833	Imd	*diptericin-A*	Fw	CCAATTCAGGAAGTGGAACC	56	Antimicrobial peptide
			Rv	TGTTGATGGGTAGCTCCAAA	56	
AAEL014148	Imd	*dredd*	Fw	GTGGCTGTTATGCGAGAAGA	60	Initiatior caspase, cleavage of REL2
			Rv	AGCGTAGTTCTGCCTGAGGT	60	
AAEL001932	Imd	*FADD*	Fw	GGGACCGTCGAACACTTCTT	60	Imd signal transducer
			Rv	CACTCAGCTGCATTAACCGC	60	
AAEL007768	Toll	*MyD88*	Fw	GGACTACAAGCGCTCGAACA	60	TOLL signaling cascade starter
			Rv	CTGGTTTGGTTTGCGTTCGA	60	
AAEL006571	Toll	*PELLE*	Fw	ACAACCGACGAAAACTCCGA	56	Signaling molecule - Kinase
			Rv	GCGAAGTTCTTCCCCACTGA	56	
AAEL000718	JAK/STAT	*vir-1*	Fw	GCCAAAGTCCGGTATTCTTC	60	Antiviral effector
			Rv	TTCACGAGATCGTCAAGGTAA	60	
AAEL004175	—	*rpS17*	Fw	TCCGTGGTATCTCCATCAAGCT	60	Ribosomal small subunit assembly
			Rv	CACTTCCGGCACGTAGTTGTC	60	
AE017196	—	*WD0513*	Fw	GTATCCAACAGATCTAAGC	60	
			Rv	ATAACCCTACTCATAGCTAG	60	
NC_001474.2	—	DENV	Fw	AAGGACTAGAGGTTAGAGGAGACCC	60	
			Rv	CGTTCTGTGCCTGGAATGATG	60	
			Pr	HEX-AACAGCATATTGACGCTGGGAGAGACCAGA-BHQ1		

Gene function from Flybase and Swiss-Prot.
