# Supplementary material for: The RNAi pathway plays a small part in Wolbachia-mediated blocking of dengue virus in mosquito cells
Source: Sci Rep. 2017 Mar 6;7:43847. doi: 10.1038/srep43847 (PMC5338330; doi:10.1038/srep43847)
Supplement: Supplementary Figures [file srep43847-s1.pdf]

The RNAi pathway plays a small part in *Wolbachia*-mediated blocking of dengue virus in mosquito cells

Gerard Terradas[1], D. Albert Joubert[2] and Elizabeth A. McGraw*[1]

[1]School of Biological Sciences, Monash University, Clayton VIC 3800, Melbourne, Australia

[2]Institute of Vector-borne Disease, Monash University, Clayton VIC 3800, Melbourne, Australia

*Corresponding author
School of Biological Sciences
Monash University
Clayton, Vic 3800 Australia
Ph: +61 3 9902 0167
beth.mcgraw@monash.edu

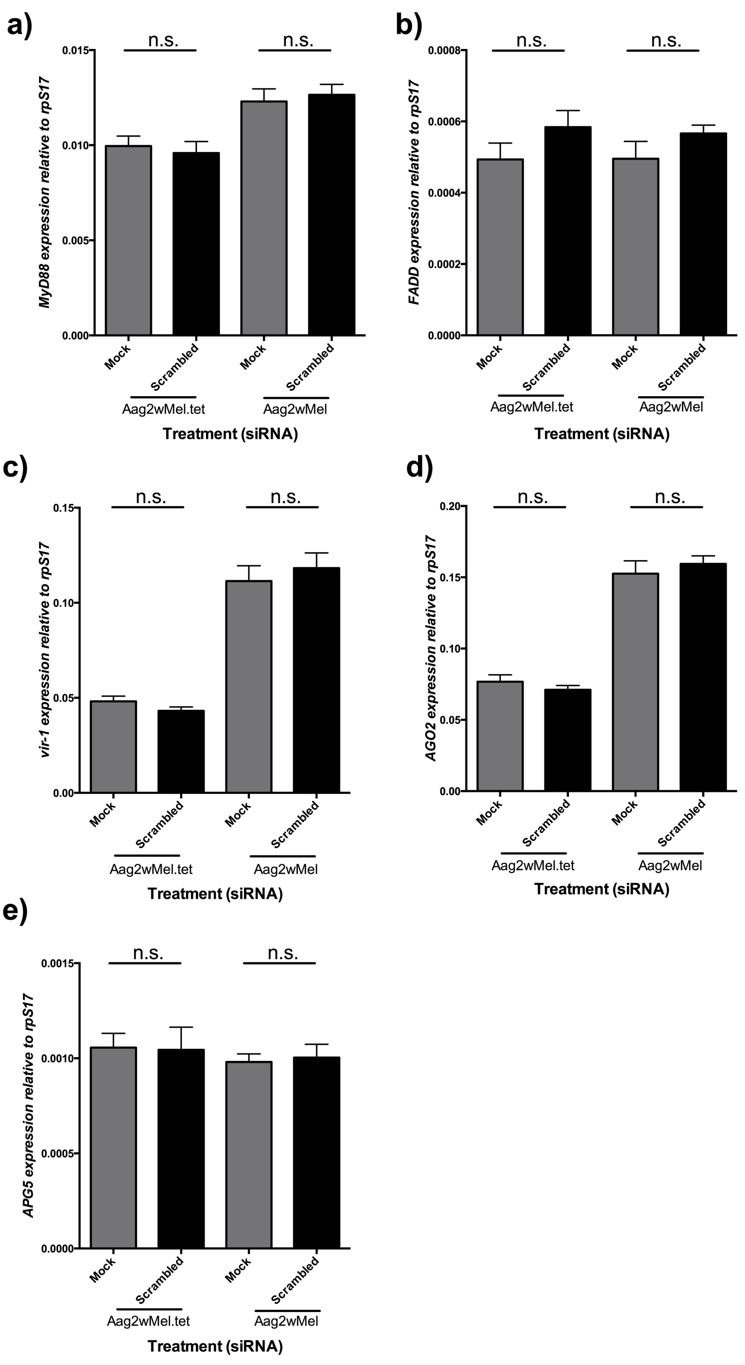

Figure S1. – The effects of scrambled siRNA (RNAiMAX + Scrambled siRNA) on the expression of each gene relative to a mock control (with RNAiMAX reagent) **(a - e). Graphs show medians with interquartile range bars** (n = 6 per treatment). Unpaired t-tests after logarithmic transformation of data revealed no significant differences.

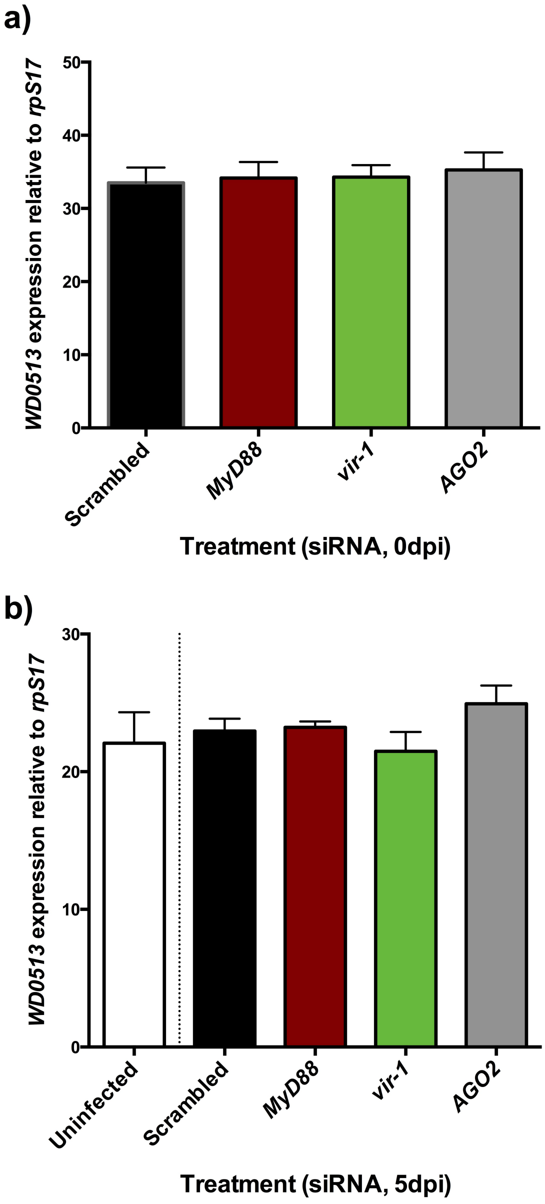

Figure S2 – *Wolbachia* levels are not affected by siRNA treatment. Relative quantification of the *Wolbachia* gene *WD0513* to *Aedes aegypti* housekeeping *rpS17* proved that *Wolbachia* levels are not modulated by the transfection of siRNA either prior to **(a)** or 5 days after DENV infection **(b)**. Unpaired t-tests after logarithmic transformation of data revealed no significant differences.

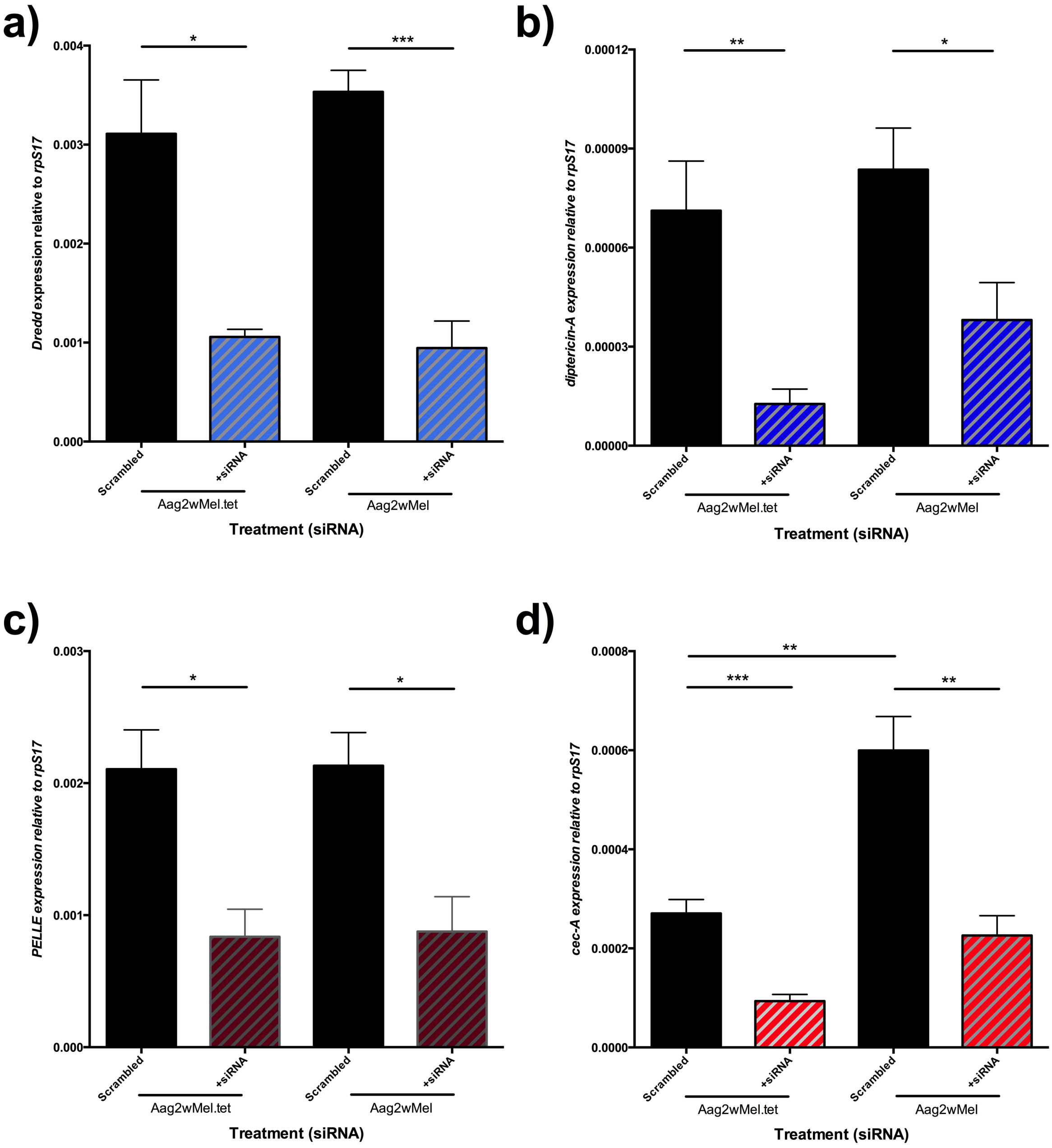

Figure S3. – After knockdown of *MyD88* and *FADD*, other genes involved downstream of **(a, b)** Toll and **(c, d)** Imd pathways are shown. Graphs show medians with interquartile range bars (n = 5 per treatment). Black columns depict scrambled controls. Significance is based on unpaired t-tests on logarithmic transformed data. *, $p<0.05$; **, $p<0.01$; ***, $p<0.001$; ****, $p<0.0001$.

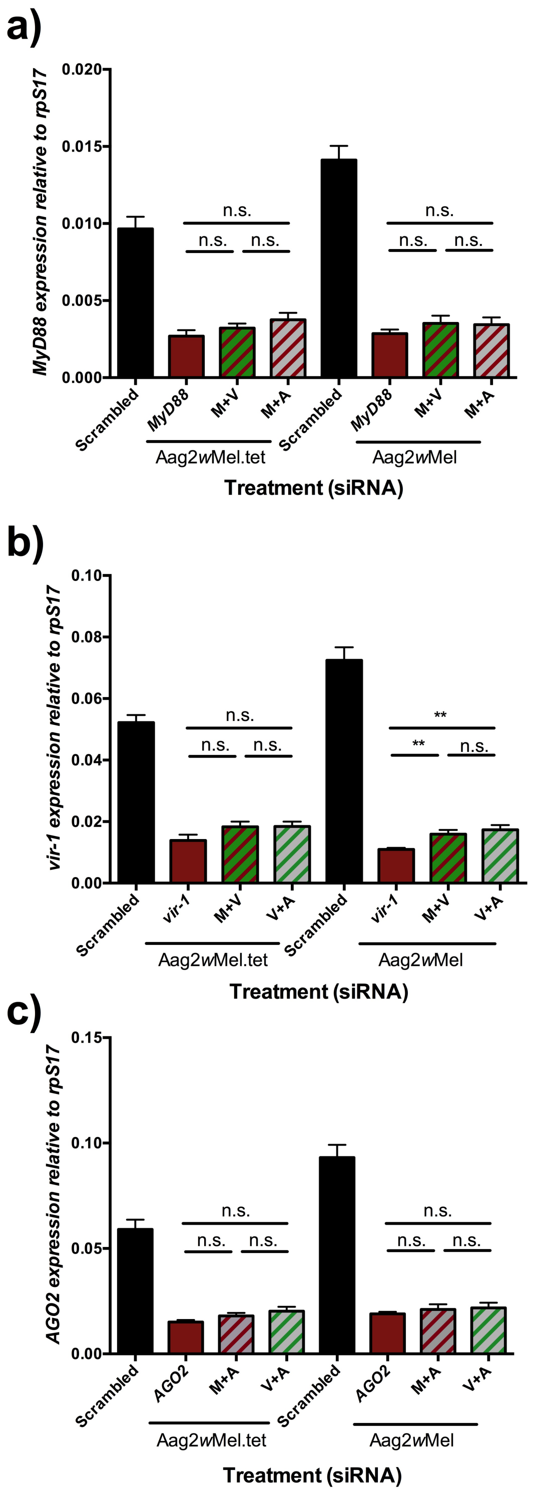

Figure S4. – Knockdown of *MyD88* (M) **(a)**, *vir-1* (V) **(b)** and *AGO2* **(c)** after targeted siRNA treatment. Graphs show medians with interquartile range bars (n = 12 per treatment). Black columns depict scrambled controls. Significance is based on logarithmic transformed data. n.s.= non-significant; **, p<0.01; ***, p<0.001.
